# Clinical manifestations of COVID‐19 differ by age and obesity status

**DOI:** 10.1111/irv.12918

**Published:** 2021-10-19

**Authors:** Wesley A. Cheng, Lauren Turner, Carolyn J. Marentes Ruiz, Melissa L. Tanaka, Zion Congrave‐Wilson, Yesun Lee, Jaycee Jumarang, Stephanie Perez, Ariana Peralta, Pia S. Pannaraj

**Affiliations:** ^1^ Division of Infectious Diseases Children's Hospital Los Angeles Los Angeles California USA; ^2^ Department of Pediatrics and Molecular Microbiology and Immunology, Keck School of Medicine University of Southern California Los Angeles California USA

**Keywords:** adolescents, children, COVID‐19, obesity, SARS‐CoV‐2

## Abstract

**Background:**

Age and obesity status are associated with severe outcomes among hospitalized individuals with COVID‐19. It remains unclear whether age and obesity are risk factors for milder COVID‐19 illness.

**Methods:**

We prospectively enrolled SARS‐CoV‐2‐exposed individuals. Participants recorded symptoms for 28 days and were tested for SARS‐CoV‐2 by reverse transcription polymerase chain reaction (RT‐PCR) and serology. Type, number, and duration of symptoms and SARS‐CoV‐2 laboratory parameters were compared by age and obesity status.

**Results:**

Of 552 individuals enrolled from June 2020 to January 2021, 470 (85.1%) tested positive for SARS‐CoV‐2 including 261 (55.5%) adults ≥18 years, 61 (13.0%) adolescents 12–17 years, and 148 (31.5%) children <12 years. Children had fewer symptoms (median 2 vs. 3, *p* < 0.001) lasting fewer days (median 5 vs. 7, *p* < 0.001) compared with adolescents/adults. Body mass index of 300 (63.8%) individuals classified with overweight or obesity (OWOB). Individuals with OWOB suffered more symptoms compared with individuals without OWOB (median 3 vs. 2, *p* = 0.037), including more cough and shortness of breath (*p* = 0.023 and 0.026, respectively). Adolescents with OWOB were more likely to be symptomatic (66.7% vs. 34.2%, *p* = 0.008) and have longer respiratory symptoms (median 7 vs. 4 days, *p* = 0.049) compared with adolescents without OWOB. Lower RT‐PCR Ct values were found in children and symptomatic individuals compared with adolescent and adults and asymptomatic individuals, respectively (*p* = 0.001 and 0.022).

**Conclusions:**

Adolescents and adults with OWOB experience more respiratory symptoms from COVID‐19 despite similar viral loads. These findings underscore the importance of vaccinating individuals with OWOB.

## 1 INTRODUCTION

Age and obesity status are both major determinants of host response to pathogens, including during severe acute respiratory syndrome coronavirus‐2 (SARS‐CoV‐2) infection.^1^, ^2^ Incidence of coronavirus disease 2019 (COVID‐19) appears to be increasing in children, paralleling adult trends.^3^ Most infected children are asymptomatic or have mild symptoms. However, hospitalization rates have been increasing among children and adolescents starting in the spring of 2021.^4^ Obesity is a prevalent comorbidity in both adults and children hospitalized with severe COVID‐19.^5^, ^6^ The likelihood of hospitalization and risk for severe COVID‐19 increases with higher body mass index (BMI) in adults may be associated with chronic inflammation disrupting immune responses and impairing pulmonary function.^7^, ^8^, ^9^ The elevated risk of complications in obese individuals was reflected in the Advisory Committee on Immunization Practices (ACIP)'s inclusion of obesity as high‐risk condition for COVID‐19 vaccine prioritization in the initial guidelines before widespread availability^10^ and the Food and Drug Administration (FDA)'s Emergency Use Authorization for receipt of an anti‐SARS‐CoV‐2 monoclonal antibody.^11^

Most published data have focused on disease presentation and prognosis of hospitalized individuals, but the majority of COVID‐19 infected individuals are managed outside of the hospital. Less data are available on COVID‐19 infected non‐hospitalized individuals with overweight or obesity (OWOB), and very few studies have focused on COVID‐19 in children with OWOB.^12^, ^13^, ^14^ This prospective study analyzes differences in symptom types and duration as well as SARS‐CoV‐2 laboratory parameters by age and obesity status in predominantly outpatient COVID‐19‐positive patients reflective of the majority of COVID‐19 cases in the general population.

## 2 MATERIALS AND METHODS

### 2.1 Study design and participants

We enrolled individuals within 2 weeks of exposure to a laboratory‐confirmed COVID‐19 household contact into the Household Exposure and Respiratory Virus Transmission and Immunity Study (HEARTS). We recruited household members of individuals who tested positive for SARS‐CoV‐2 at the Children's Hospital Los Angeles (CHLA) laboratory using a convenience recruitment strategy. Those who have specimens tested at the laboratory include symptomatic and asymptomatic staff and patients of CHLA and network outpatient clinics. Recruitment fliers were also posted at community testing sites near the hospital. At enrollment, individuals answered a questionnaire that included demographic information, comorbidities, and exposure history. Participants logged illness symptoms and symptom severity in a daily symptom diary for 28 days; study staff followed up on the symptom diary over the phone or at in‐person follow‐up visits every 3–7 days. Parents/guardians completed questionnaires and recorded symptoms for children unable to record for themselves. COVID‐19‐associated symptoms were defined (and grouped) as experiencing at least one of the following: fever, chills, headache, fatigue, muscle aches (constitutional); runny nose, congestion, sore throat, cough, shortness of breath, wheeze (respiratory); altered smell, altered taste (neurologic); vomiting, diarrhea, or abdominal pain (gastrointestinal). Data was recorded using Research Electronic Data Capture software (REDCap Consortium, Vanderbilt, Tennessee, USA). The study was approved by the Institutional Review Board at Children's Hospital Los Angeles. Informed consent was obtained from all participants.

Participants presented for nasopharyngeal (NP) swab collection, performed by trained study staff every three to 7 days until two consecutive negative SARS‐CoV‐2 real‐time reverse transcription polymerase chain reaction (RT‐PCR) results were obtained in the entire household or they reached their sixth visit. Saliva samples were also collected at each of these visits from able individuals. Blood was collected at the first visit and a convalescent visit at least 4 weeks after all household members tested negative. Height and weight were measured at the first visit. The Children's BMI Tool for Schools from the CDC was used to calculate BMI percentiles factoring in age and sex for children and teens that are 2–19 years old.^15^ For individuals 20 years or older, BMI was interpreted using standard weight status categories as follows: BMI < 18.5 underweight, 18.5–24.9 normal weight, 25–29.9 overweight, ≥30 obese.^15^ BMI charts are not recommended for clinical use in children under 2 years of age.

### 2.2 SARS‐CoV‐2 RT‐PCR

We tested for SARS‐CoV‐2 using the CDC protocol approved by the Food and Drug Administration for Emergency Use Authorization.^16^ Briefly, total nucleic acid was extracted from 200‐μl NP swab samples using the QIAamp Viral RNA Mini Kit (QIAGEN, Valencia, CA, USA) and eluted to 50 μl of total nucleic acid. RT‐PCR was performed using primers and probes that targeted the N1, N2 and RnaseP (RNP, internal control) genes (IDT, Coralville, IW) with 1‐Step Taqpath Master Mix (Thermo Fisher, Carlsbad, CA, USA) on QuantStudio 5 (Applied Biosystem, Carlsbad, CA, USA). A positive result was defined as cycle threshold (Ct) value less than 40 for both N1 and N2. A valid result for SARS‐CoV‐2 detection was determined by RNP using a cut‐off of Ct value <32. An inconclusive result was defined as either N1 or N2 gene detected only with RNP detection.

### 2.3 SARS‐CoV‐2 serology

Serum SARS‐CoV‐2 receptor binding domain (RBD) and spike IgG antibody was measured using an ELISA as previously described.^17^ A positive cut‐off OD_490_ value of 0.2 was used for RBD IgG detection based on the published protocol and the mean of the negative control values plus three standard deviations (SDs) from 20 blood samples collected between 2017 and 2019. IgG against the spike antigen was used to confirm RBD IgG positivity. Area under the curve (AUC) values were calculated using IgG OD_490_ values from 5 serial diluted samples (1:100–8100) tested against the spike antigen.

### 2.4 Statistics

COVID‐19 onset was defined as the earlier date between the first symptom presentation and first PCR positivity. For comparisons of the PCR Ct values between individuals, we used the lowest RT‐PCR Ct value from NP swabs obtained within the first 7 days from COVID‐19 onset. For the length of PCR positivity calculation, subjects were included if they had a PCR positivity end date, defined as the last date of a positive test before a PCR negative or inconclusive result. For comparison of SARS‐CoV‐2 antibody levels, we used the highest spike protein‐specific IgG AUC between 14 and 90 days of COVID‐19 onset. For comparisons of obesity categories, overweight and obese (OWOB) were grouped together and compared with non‐OWOB individuals.

Comparisons of categorical variables were calculated using Pearson's chi‐squared or Fisher's exact test as appropriate. Mann–Whitney *U* or Kruskal–Wallis tests were used for comparison of non‐parametric data. Independent sample *t* tests were used for comparison of normally distributed continuous variables or for log‐transformed data. Multiple logistic regression using the backward selection method was used to determine predictors of symptoms. Factors with *p* < 0.10 in the univariate analysis were included in the multivariable analysis. Statistical analyses were performed using SPSS Version 27.0 (IBM Corp., Armonk, NY, USA) and RStudio Version 1.3.1093 (RStudio Inc., Boston, MA, USA). All tests were two‐tailed with *p* < 0.05 considered significant.

## 3 RESULTS

We enrolled 560 individuals from June 17, 2020 to January 31, 2021; all had exposure to a household member with laboratory‐confirmed SARS‐CoV‐2 infection. Of those, 4 were excluded because they did not present for NP PCR testing; 4 others were excluded due to prior documented COVID‐19 infection greater than 3 months before enrollment. The remaining 552 individuals underwent NP swab testing for PCR detection of SARS‐CoV‐2; 399 (72.3%) also provided saliva for PCR testing. Blood was obtained from 476 (86.2%) participants at enrollment and from 283 (51.3%) at a convalescent visit. A total of 470 (85.1%) participants had laboratory‐confirmed SARS‐CoV‐2 infection as defined by a positive PCR result via NP or saliva sampling and/or positive serology. Five patients had inconclusive PCR results with negative serology at enrollment and no available convalescent serology; they were excluded from the analyses due to inability to classify them as case or non‐case. Therefore, we analyzed 547 laboratory‐confirmed COVID‐19 cases and non‐cases.

Participant ages ranged from 1 month to 84 years, including 308 (56.3%) adults ≥18 years old and 239 (43.7%) children <18 years old. Ethnicity in the cohort was characterized by a strong Hispanic/Latinx predominance, which reflects the surrounding community served by our institution. Among all enrolled subjects, 107 (19.6%) reported an underlying medical condition; asthma, diabetes, and cardiovascular conditions were most common. BMI of all subjects ≥2 years old averaged 25.9 ± SD 6.0 kg/m^2^. Nearly two‐thirds (307 [62.0%]) were with OWOB, including a large proportion of enrolled adults (229 [74.6%]) and children (116 [61.7%]). Characteristics of the 547 laboratory‐confirmed COVID‐19 cases and non‐cases are shown in Table 1. The characteristics more common in COVID‐19 cases compared with non‐cases were Hispanic/Latinx ethnicity and lower household income (*p* = 0.001 and 0.044, respectively).

**TABLE 1:** Characteristics^a^ of COVID‐19 cases and non‐cases

Characteristic	Cases *n* = 470 (%)	Non‐cases *n* = 77 (%)	*p* value
Sex			0.15
Male	209 (44.5)	41 (53.2)	
Female	261 (55.5)	36 (46.8)	
Age group			0.23
≤12 years old	148 (31.5)	20 (26.0)	
13–17 years old	61 (13.0)	10 (13.0)	
18–29 years old	103 (21.9)	12 (15.6)	
30–54 years old	136 (28.9)	28 (36.4)	
≥55 years old	22 (4.7)	7 (9.1)	
Ethnicity			0.001
Hispanic	444 (94.5)	65 (84.4)	
Not Hispanic	26 (5.6)	12 (15.6)	
Race			
White	455 (96.6)	71 (93.4)	0.079
African American	1 (0.2)	2 (2.6)	
Asian	11 (2.3)	3 (3.9)	
Multiple	4 (0.8)	0 (0)	
BMI category			0.66
Underweight	4 (0.9)	1 (1.4)	
Normal	120 (28.3)	25 (35.2)	
Overweight	136 (32.1)	20 (28.2)	
Obese	164 (38.7)	25 (35.2)	
Underlying conditions^b^	89 (18.9)	18 (23.4)	0.36
Allergies	18 (3.8)	2 (2.6)	
Asthma or other chronic lung disease	37 (7.9)	8 (10.5)	
Heart condition	28 (5.9)	5 (6.6)	
Diabetes	24 (5.1)	3 (3.9)	
Renal disease	4 (0.8)	1 (1.3)	
Liver disease	1 (0.2)	0 (0)	
Cancer or other immunosuppression	3 (0.6)	2 (2.6)	
Neurologic/genetic conditions	9 (1.9)	2 (2.6)	
Currently pregnant	5 (1.1)	0 (0)	
Taking long term prescription medication/device^c^	54 (11.5)	11 (14.3)	0.48
Smoker	6 (7.8)	20 (4.3)	0.18
Smoker in the household	41 (8.7)	4 (5.2)	0.30
Income			0.044
Less than $20,000	114 (24.3)	17 (22.1)	
$20,000 to $34,999	208 (44.3)	25 (32.5)	
$35,000 to $49,999	58 (12.3)	9 (11.7)	
$50,000 to $74,999	46 (9.8)	14 (18.2)	
$75,000 to 99,999	24 (5.1)	4 (5.2)	
Over $100,000	20 (4.3)	8 (10.4)	
Traveled in the past month	6 (1.3)	0 (0)	1.0

*Note:* The proportions of each group within cases versus non‐cases are compared.

^a^ All characteristics are self‐reported except body mass index (BMI) for which height and weight were measured.

^b^ Does not include obesity.

^c^ Device includes continuous positive airway pressure (CPAP) machine.

Of 470 subjects with confirmed COVID‐19, 262 (55.7%) were symptomatic and 208 (44.3%) were asymptomatic. Adults were more likely to be symptomatic compared with children (59.8 vs. 50.7%, *p* = 0.05). More individuals with OWOB were symptomatic compared to individuals without OWOB (59.0 vs. 46.8%, p = 0.021). In a multivariable analysis, only higher BMI and higher household income were associated with higher odds of self‐reporting symptoms (odds ratio [OR] 1.05 and 1.51, *p* = 0.002 and <0.001, respectively) (Table 2). No participants received an anti‐SARS‐CoV‐2 monoclonal antibody or other outpatient treatment. Six subjects were hospitalized at the time of COVID‐19 positivity, but only three hospitalizations (ages: 13, 17, and 45 years old; BMI status: obese, normal, overweight, respectively) were secondary to COVID‐19‐related complications.

**TABLE 2:** Factors associated with increased odds of symptomatic versus asymptomatic infection in 470 laboratory‐confirmed COVID‐19 cases

Characteristics^a^	Symptomatic *n* = 262/470 (55.7%)	Univariate analysis		Multivariable analysis	
		Odds ratio (95% CI)	*p* value	Odds ratio (95% CI)	*p* value
Age^b^		1.01 (0.10–1.02)	0.21	—	—
≤12 years old	74 (50.0)	—	—	—	—
13–17 years old	32 (52.5)	—	—	—	—
18–29 years old	58 (56.3)	—	—	—	—
30–54 years old	85 (62.5)	—	—	—	—
≥55 years old	13 (59.1)	—	—	—	—
Male sex	113 (54.1)	0.89 (0.61–1.28)	0.51	—	—
Hispanic/Latinx ethnicity	244 (55.0)	0.954 (0.23–1.27)	0.16	—	—
Race					
White	250 (55.1)	—	≥0.99	—	—
African American	0 (0)	—	≥0.99	—	—
Asian	8 (72.7)	—	≥0.99	—	—
Multiple	4 (100)	—	—	—	—
BMI	—	1.05 (1.02–1.09)	0.002	1.05 (1.01–1.08)	0.005
Underweight	0 (0)	—	—	—	—
Normal	58 (48.3)	—	—	—	—
Overweight	80 (58.8)	—	—	—	—
Obese	97 (59.1)	—	—	—	—
Underlying conditions^c^	57 (64.0)	0.65 (0.406–1.05)	0.081	0.63 (0.38–1.06)	0.079
Taking long term prescription medication/device^d^	23 (42.6)	0.93 (0.52–1.64)	0.79	—	—
Smoker	11 (55.0)	1.03 (0.42–2.54)	0.95	—	—
Smoker in household	21 (51.2)	1.22 (0.64–2.32)	0.54	—	—
Household income^b^		1.51 (1.29–1.77)	<0.001	1.50 (1.28–1.77)	<0.001
Less than $20,000	39 (34.2)	—	—	—	—
$20,000 to $34,999	125 (60.1)	—	—	—	—
$35,000 to $49, 999	31 (53.4)	—	—	—	—
$50,000 to $74,999	34 (73.9)	—	—	—	—
$75,000 to 99,999	13 (54.2)	—	—	—	—
over $100,000	20 (100)	—	—	—	—

Abbreviations: BMI, body mass index; CI, confidence interval

^a^ All characteristics are self‐reported except BMI for which height and weight were measured.

^b^ Age, BMI, and household income entered into logistic regression model as continuous or ordinal variables; categories are shown for reference only.

^c^ Does not include obesity.

^d^ Device includes continuous positive airway pressure (CPAP) machine.

**FIGURE 1:**
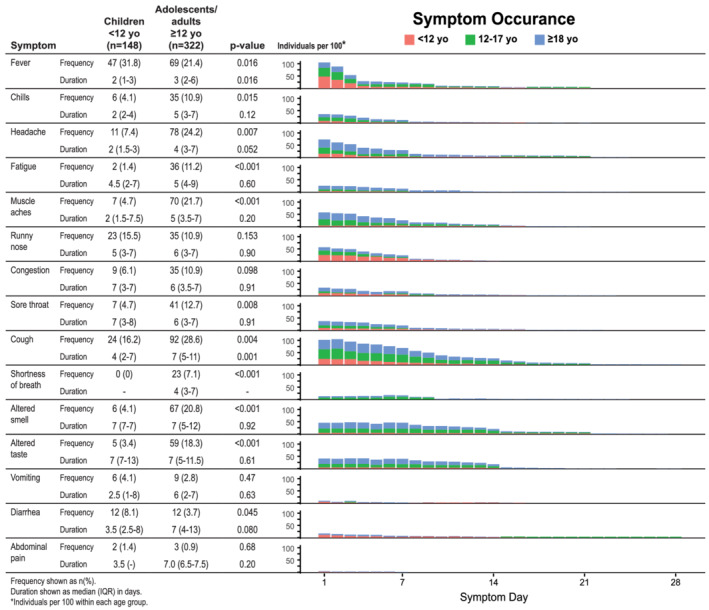
Symptoms in adolescents and adults ≥12 years old (yo) versus children <12 yo. Children <12 yo experience more fever and diarrhea, but adolescents and adults have more chills, headache, fatigue, muscle aches, sore throat, cough, shortness of breath, and altered smell and taste (all *p* < 0.05). Fever and cough lasted longer in adults and adolescents (*p* = 0.016 and 0.001, respectively)

Symptomatic individuals reported a median of 3 (interquartile [IQR] range 2–4) symptoms that lasted a median of 7 (IQR 4–11) days (Figure 1). For almost all symptoms, adolescents aged 12–17 years old had symptom frequency, distribution, and duration similar to adults ≥18 years old; the exceptions were headache, loss of taste and loss of smell, with incidence between those of adults and younger children <12 years old. Therefore, we grouped adolescents with adults for comparison with younger children <12 years old (Figure 1). Younger children had fewer symptoms (median 2 [IQR 1–3] vs. 3 [IQR 2–5], *p* < 0.001) that lasted fewer days (median 5 [IQR 3–7] vs. 7 [IQR 5–13], *p* < 0.001) compared with adolescents and adults. Younger children had more fever and diarrhea, but adults and adolescents reported more fatigue, sore throat, cough, shortness of breath, headache, and altered taste and smell (all *p* < 0.05). Fever and cough lasted longer in adolescents and adults compared with younger children (*p* = 0.016 and 0.001, respectively). Of those symptomatic, adolescents and adults (individuals ≥12 years old) reported moderate or severe symptoms more often than children <12 years old (18.6% vs. 8.6%, *p* = 0.048).

**FIGURE 2:**
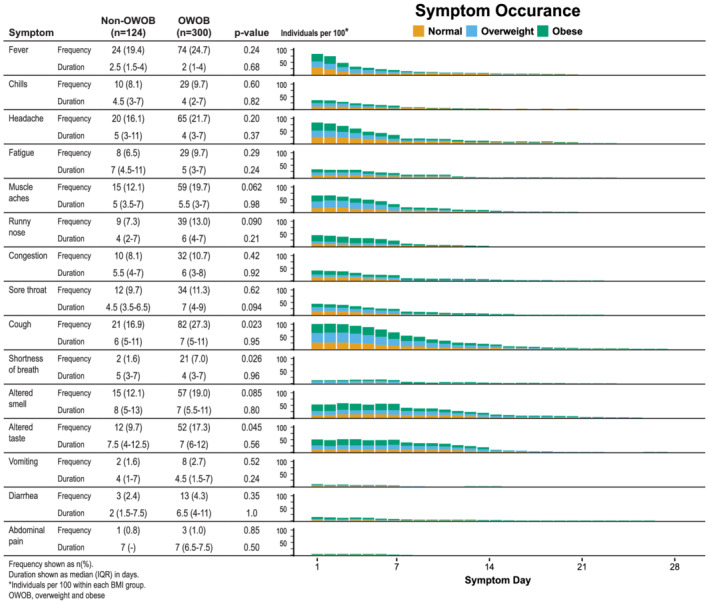
Symptoms in individuals with overweight and obese (OWOB) compared with individuals without OWOB. Individuals with OWOB experienced more cough, shortness of breath, and altered taste compared with individuals without OWOB (*p* = 0.023, 0.026, and 0.045, respectively). The duration of symptoms was similar between the two groups

Individuals with OWOB suffered from more symptoms compared with individuals without OWOB (median 3 [IQR 2–4] vs. 2 [IQR 1–3], p = 0.037) (Figure 2). Individuals with OWOB experienced significantly more cough (82 [27.3%] vs. 21 [16.9%], *p* = 0.023), shortness of breath (21 [7.0%] vs. 2 [1.6%], *p* = 0.026), and altered taste (52 [17.3] vs. 12 [9.7%], 0.045). The frequency of reported moderate or severe symptoms were similar in the two groups (17.9% vs. 19.5%, *p* = 0.31). The median symptom duration was also similar between the two groups (median 7 [IQR 4–11] vs. 6.5^4^, ^5^, ^6^, ^7^, ^8^, ^9^, ^10^, ^11^ days, *p* = 0.39).

**FIGURE 3:**
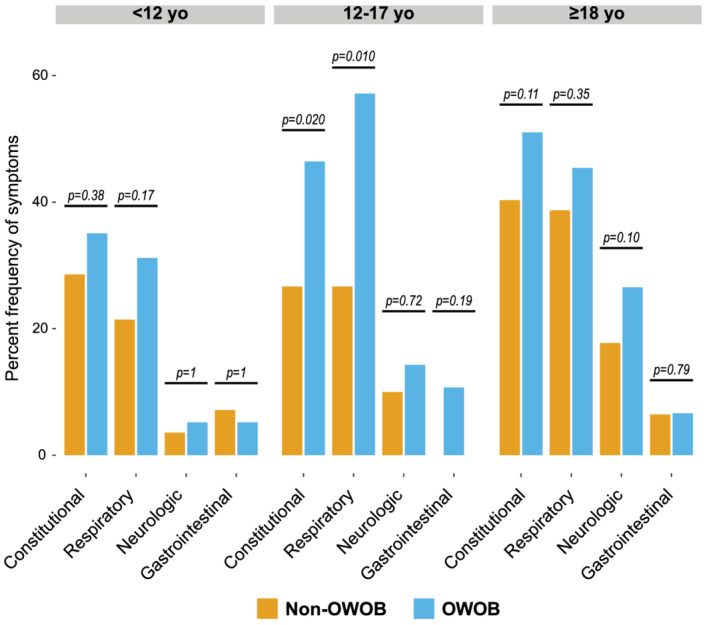
COVID‐19 associated symptoms by age group and obesity status. Adolescents 12–17 years old (yo) with overweight and obese (OWOB) experience more constitutional and respiratory symptoms compared with adolescents without OWOB (*p* = 0.020 and 0.010, respectively)

We further examined if experiencing obesity impacted the severity of illness by age group in individuals with known BMI status (Figure 3). In the 120 younger children <12 years old, there were no significant differences in the presence, number, or duration of symptoms in children of different BMI categories. Evaluation of the 68 adolescents, however, revealed significantly more frequent presence of symptoms among adolescent with OWOB compared with those without (20 [66.7%] vs 13 [34.2%], *p* = 0.008), including constitutional symptoms (15 [50.0%] vs. 8 [21.1%], *p* = 0.012) and respiratory symptoms (16 [53.3%] vs. 8 [21.1%], *p* = 0.006). Respiratory symptoms lasted longer in adolescents with OWOB (median 7 [IQR 2–9] vs. 4 [IQR 1–6] days, p = 0.049). Duration of constitutional, neurologic and gastrointestinal symptoms were similar. In the 307 adults, only presence of shortness of breath differed between individuals with OWOB compared with those without (19 [8.3%] vs. 1 [1.3%], p = 0.030). Duration was similar for all symptoms.

Differences in laboratory parameters were evaluated by symptom presentation, age group, and obesity status (Table 3). In a comparison of the lowest RT‐PCR Ct values from NP swabs obtained within the first 7 days of COVID‐19 onset, symptomatic individuals and younger children <12 years had lower Ct values compared with asymptomatic participants and those in the adolescent and adult age groups, respectively (*p* = 0.001 and 0.022, Table 3). No differences were observed in individuals with OWOB compared with individuals without OWOB. Duration of PCR positivity was longer in symptomatic individuals compared with asymptomatic individuals (17.5 vs. 8.4 days *p* = 0.014), but no differences in duration was found by age or obesity status. COVID‐19 spike‐specific IgG AUC did not differ by presence of symptoms, age or obesity status.

**TABLE 3:** Laboratory parameters associated with symptoms, age, and obesity status

Characteristic (*n*)	Laboratory parameter	*p* value
RT‐PCR Ct value^a^		
Symptomatic (141)	23.8	0.001
Asymptomatic (92)	27.3	
<12 years old (93)	23.9	0.022
≥12 years old (140)	26.0	
Non‐obese/overweight (71)	26.4	0.19
Obese/overweight (135)	25.1	
Duration of PCR detection (days)^b^		
Symptomatic (204)	17.5	0.014
Asymptomatic (101)	8.4	
years old (81)	15.1	0.84
≥12 years old (224)	14.3	
Non‐obese/overweight (90)	16.1	0.27
Obese/overweight (192)	12.7	
COVID‐19 spike protein IgG (AUC)^c^		
Symptomatic (55)	393.7	0.60
Asymptomatic (26)	333.1	
years old (17)	333.1	0.19
≥12 years old (64)	393.7	
Non‐obese/overweight (18)	392.5	0.81
Obese/overweight (58)	358.8	

Abbreviation: AUC, area under the curve; RT‐PCR, reverse transcription polymerase chain reaction.

^a^ Lowest RT‐PCR Ct value from nasopharyngeal swabs obtained within the first 7 days from COVID‐19 onset. Individuals with specimen collection after 7 days were excluded.

^b^ Participants were included if they had PCR positivity end date, defined as the last date of a positive test before a PCR negative or inconclusive result.

^c^ Highest spike protein‐specific IgG AUC between 14 and 90 days of COVID‐19 onset. Specimens obtained out of this range were excluded.

## 4 DISCUSSION

Nearly two‐thirds of our study participants were with overweight or obesity, reflective of worsening obesity trends in the United States and worldwide.^18^, ^19^ The effect of the COVID‐19 pandemic's lockdown on lifestyle behaviors including increase intake of high fat and high caloric diets, increased screen time, and less physical activities may contribute further to the obesity epidemic.^20^, ^21^ This phenomenon is especially alarming as COVID‐19 and its variants continue to circulate. In our predominantly outpatient cohort, COVID‐19 infected individuals with OWOB experienced more symptoms, especially respiratory symptoms of cough and shortness of breath, compared with individuals without OWOB. These findings are in line with findings of increased risk of severe outcomes in COVID‐19 hospitalized individuals with OWOB, including ICU admission, invasive mechanical ventilation, and death.^2^, ^5^

While OWOB status in pre‐pubescent children <12 years of age did not affect clinical presentation of COVID‐19, adolescents with OWOB experienced more frequent and longer duration of respiratory symptoms. Overall symptom frequency, duration, and self‐reported severity levels in adolescents were more similar to adults. Adolescents had more symptoms at higher self‐reported severity that lasted longer than younger children <12 years old. Hospitalization rates for COVID‐19 infected adolescents in the United States increased in the spring of 2021, and nearly a third required admission to the intensive care unit.^4^ The report noted that the most common risk factor for hospitalization was obesity in 35.8% of those hospitalized. Furthermore, approximately two‐thirds of adolescents hospitalized with COVID‐19 were Hispanic or non‐Hispanic Black in the population‐based surveillance study. Obesity disproportionately impacts Hispanic and non‐Hispanic Black communities and persons from low‐income households, who are also more likely to suffer worse disease outcomes.^2^, ^22^, ^23^ Most of our study participants were from Hispanic and low‐income households.

Interestingly, 65% of individuals from households in the lowest income bracket (household incomes under $20,000) reported having no COVID‐19 associated symptoms, whereas 100% of those in the highest income bracket (>$100,000) reported symptoms. Differences in symptoms by income level were found to be significant. Health inequities and social pressures might be contributing factors to lack of reporting by individuals from low‐income households. Poor access to healthcare, stigma, mistrust, language barriers, work conditions, and financial burden can all hinder accurate self‐reporting of symptoms or symptom severity in patients from communities where these issues are prominent.^24^ Downplaying symptoms may lead to a delay in care, further exacerbating the disparities in COVID‐19 infection outcomes.

Our data support the finding of predominantly mild to asymptomatic SARS‐CoV‐2 infections in younger children <12 years old. The significantly lower COVID‐19 symptom frequency in the pre‐school and elementary school‐aged children suggests that they are more likely to have undetected or overlooked infections. To what extent children can transmit SARS‐CoV‐2 is still under investigation as many communities and schools reopen.^25^, ^26^, ^27^ Although the younger children in our study had higher viral loads suggested by lower PCR Ct values, they were less likely to have cough as a method of spreading the virus. Even if they were symptomatic, the duration of symptoms was shorter compared with adolescents and adults. Overall, symptomatic children and adults were more likely to have higher viral loads and have detectable SARS‐CoV‐2 by RT‐PCR for a longer duration compared with asymptomatic individuals.

The data should be interpreted with the consideration of some limitations. All symptom data were self‐reported in a daily symptom diary and can be subjective and subject to recall bias if symptoms began prior to enrollment. Our study included households with young children who relied on parents or guardians to report the symptom data of their children. Young children are likely unable to verbalize and identify some symptoms including headache and loss of taste and smell. The study included a smaller number of adolescents compared with other age groups. We may not have captured all confounding variables associated with OWOB.

Our findings underscore clinical and public health needs for individuals with OWOB, particularly adolescents and adults with OWOB, in the context of COVID‐19. The data may inform primary care physicians on how to counsel individuals with OWOB regarding expected severity and duration of symptoms. Highly effective SARS‐CoV‐2 vaccines currently being distributed are expected to mitigate COVID‐19 spread in the general population. We did not see differences in antibody response to natural SARS‐CoV‐2 infection by presence of symptoms, age, or obesity status. The COVID‐19 vaccines are highly efficacious and elicit consistent immune responses in people with and without obesity,^28^ thus ameliorating preventable complications in an at‐risk population.

## AUTHOR CONTRIBUTIONS

**Wesley A. Cheng:** Data curation; formal analysis. **Lauren Turner:** Data curation; formal analysis. **Carolyn J. Marentes Ruiz:** Data curation. **Melissa L. Tanaka:** Data curation. **Zion Congrave‐Wilson:** Data curation. **Yesun Lee:** Data curation. **Jaycee Jumarang:** Data curation. **Stephanie Perez:** Data curation. **Ariana Peralta:** Data curation. **Pia Pannaraj:** Conceptualization‐Lead; Formal analysis‐Lead; Funding acquisition‐Lead; Supervision‐Lead; Writing‐original draft‐Lead; Writing‐review & editing‐Lead.

## FUNDING INFORMATION

This work was funded by NIH/NIAID U01AI144616‐02S1.

## CONFLICTS OF INTEREST

P. S. P. receives research funding from AstraZeneca and Pfizer for unrelated non‐COVID‐19 studies. She also has received consultant fees from Sanofi‐Pasteur and Seqirus. All other authors have no conflicts of interest to report.

5

### PEER REVIEW

The peer review history for this article is available at https://publons.com/publon/10.1111/irv.12918.

## References

[1] Castagnoli R , Votto M , Licari A , et al. Severe acute respiratory syndrome coronavirus 2 (SARS‐CoV‐2) infection in children and adolescents: a systematic review. JAMA Pediatr. 2020;174(9):882‐889. 32320004 10.1001/jamapediatrics.2020.1467

[2] Kompaniyets L , Goodman AB , Belay B , et al. Body mass index and risk for COVID‐19‐related hospitalization, intensive care unit admission, invasive mechanical ventilation, and death—United States, March–December 2020. MMWR Morb Mortal Wkly Rep. 2021;70(10):355‐361. 33705371 10.15585/mmwr.mm7010e4 PMC7951819

[3] Leidman E , Duca LM , Omura JD , Proia K , Stephens JW , Sauber‐Schatz EK . COVID‐19 trends among persons aged 0–24 years—United States, March 1–December 12, 2020. MMWR Morb Mortal Wkly Rep. 2021;70(3):88‐94. 33476314 10.15585/mmwr.mm7003e1 PMC7821770

[4] Havers FP , Whitaker M , Self JL , et al. Hospitalization of adolescents aged 12–17 years with laboratory‐confirmed COVID‐19—COVID‐NET, 14 States, March 1, 2020–April 24, 2021. MMWR Morb Mortal Wkly Rep. 2021;70(23):851‐857. 34111061 10.15585/mmwr.mm7023e1 PMC8191866

[5] Richardson S , Hirsch JS , Narasimhan M , et al. Presenting characteristics, comorbidities, and outcomes among 5700 patients hospitalized with COVID‐19 in the New York City area. JAMA. 2020;323(20):2052‐2059. 32320003 10.1001/jama.2020.6775 PMC7177629

[6] Zachariah P , Johnson CL , Halabi KC , et al. Epidemiology, clinical features, and disease severity in patients with coronavirus disease 2019 (COVID‐19) in a children's hospital in New York City, New York. JAMA Pediatr. 2020;174(10):e202430. 32492092 10.1001/jamapediatrics.2020.2430 PMC7270880

[7] Hoong CWS , Hussain I , Aravamudan VM , Phyu EE , Lin JHX , Koh H . Obesity is associated with poor COVID‐19 outcomes: a systematic review and meta‐analysis. Horm Metab Res. 2021;53(2):85‐93. 33395706 10.1055/a-1326-2125

[8] Hamer M , Gale CR , Kivimaki M , Batty GD . Overweight, obesity, and risk of hospitalization for COVID‐19: a community‐based cohort study of adults in the United Kingdom. Proc Natl Acad Sci U S A. 2020;117(35):21011‐21013. 32788355 10.1073/pnas.2011086117 PMC7474583

[9] Du Y , Lv Y , Zha W , Zhou N , Hong X . Association of body mass index (BMI) with critical COVID‐19 and in‐hospital mortality: a dose‐response meta‐analysis. Metabolism. 2021;117:154373. 32949592 10.1016/j.metabol.2020.154373 PMC7493748

[10] Oliver SE , Gargano JW , Marin M , et al. The advisory committee on immunization practices' interim recommendation for use of Pfizer‐BioNTech COVID‐19 vaccine—United States, December 2020. MMWR Morb Mortal Wkly Rep. 2020;69(50):1922‐1924. 33332292 10.15585/mmwr.mm6950e2 PMC7745957

[11] National Institutes of Health . Anti‐SARS‐CoV‐2 monoclonal antibodies August 4, 2021 2021. Available at: https://www.covid19treatmentguidelines.nih.gov/therapies/anti‐sars‐cov‐2‐antibody‐products/anti‐sars‐cov‐2‐monoclonal‐antibodies/. Accessed August 27, 2021.

[12] Kim L , Whitaker M , O'Halloran A , et al. Hospitalization rates and characteristics of children aged <18 years hospitalized with laboratory‐confirmed COVID‐19—COVID‐NET, 14 states, March 1–July 25, 2020. MMWR Morb Mortal Wkly Rep. 2020;69(32):1081‐1088. 32790664 10.15585/mmwr.mm6932e3 PMC7440125

[13] Taheri L , Gheiasi SF , Taher M , Basirinezhad MH , Shaikh ZA , Dehghan NN . Clinical features of COVID‐19 in newborns, infants, and children: a systematic review and meta‐analysis. Compr Child Adolesc Nurs. 2021;1‐19. 10.1080/24694193.2021.1930288 34125643

[14] Fernandes DM , Oliveira CR , Guerguis S , et al. Severe acute respiratory syndrome coronavirus 2 clinical syndromes and predictors of disease severity in hospitalized children and youth. J Pediatr. 2021;230:23‐31. e10 33197493 10.1016/j.jpeds.2020.11.016 PMC7666535

[15] Centers for Disease Control and Prevention . Healthy weight, nutrition and physical activity September 17, 2020. Available at: https://www.cdc.gov/healthyweight/assessing/index.html. Accessed May 19, 2021.

[16] Centers for Disease Control and Prevention . CDC 2019‐novel coronavirus (2019‐nCoV) real‐time RT‐PCR diagnostic panel 2020. Available at: https://www.fda.gov/media/134922/download. Accessed February 1, 2020.

[17] Stadlbauer D , Amanat F , Chromikova V , et al. SARS‐CoV‐2 seroconversion in humans: a detailed protocol for a serological assay, antigen production, and test setup. Curr Protoc Microbiol. 2020;57(1):e100. 32302069 10.1002/cpmc.100 PMC7235504

[18] Hales CM , Fryar CD , Carroll MD , Freedman DS , Ogden CL . Trends in obesity and severe obesity prevalence in US youth and adults by sex and age, 2007–2008 to 2015–2016. JAMA. 2018;319(16):1723‐1725. 29570750 10.1001/jama.2018.3060 PMC5876828

[19] Sanchis‐Gomar F , Lavie CJ , Mehra MR , Henry BM , Lippi G . Obesity and outcomes in COVID‐19: when an epidemic and pandemic collide. Mayo Clin Proc. 2020;95(7):1445‐1453. 32622449 10.1016/j.mayocp.2020.05.006 PMC7236707

[20] Pietrobelli A , Pecoraro L , Ferruzzi A , et al. Effects of COVID‐19 lockdown on lifestyle behaviors in children with obesity living in Verona, Italy: a longitudinal study. Obesity (Silver Spring). 2020;28(8):1382‐1385. 32352652 10.1002/oby.22861 PMC7267384

[21] Jenssen BP , Kelly MK , Powell M , Bouchelle Z , Mayne SL , Fiks AG . COVID‐19 and changes in child obesity. Pediatrics. 2021;147(5):e2021050123. 33653879 10.1542/peds.2021-050123

[22] Ogden CL , Carroll MD , Fakhouri TH , et al. Prevalence of obesity among youths by household income and education level of head of household—United States 2011–2014. MMWR Morb Mortal Wkly Rep. 2018;67(6):186‐189. 29447142 10.15585/mmwr.mm6706a3 PMC5815488

[23] Weaver RG , Brazendale K , Hunt E , Sarzynski MA , Beets MW , White K . Disparities in childhood overweight and obesity by income in the United States: an epidemiological examination using three nationally representative datasets. Int J Obes (Lond). 2019;43:1210‐1222. 30718822 10.1038/s41366-019-0331-2 PMC11460982

[24] Macias Gil R , Marcelin JR , Zuniga‐Blanco B , Marquez C , Mathew T , Piggott DA . COVID‐19 pandemic: disparate health impact on the Hispanic/Latinx population in the United States. J Infect Dis. 2020;222(10):1592‐1595. 32729903 10.1093/infdis/jiaa474 PMC7454709

[25] Posfay‐Barbe KM , Wagner N , Gauthey M , et al. COVID‐19 in children and the dynamics of infection in families. Pediatrics. 2020;146(2):e20201576. 32457213 10.1542/peds.2020-1576

[26] Zhu Y , Bloxham CJ , Hulme KD , et al. A meta‐analysis on the role of children in severe acute respiratory syndrome Coronavirus 2 in household transmission clusters. Clin Infect Dis. 2021;72(12):e1146‐e1153. 33283240 10.1093/cid/ciaa1825 PMC7799195

[27] Yonker LM , Neilan AM , Bartsch Y , et al. Pediatric severe acute respiratory syndrome coronavirus 2 (SARS‐CoV‐2): clinical presentation, infectivity, and immune responses. J Pediatr. 2020;227:45‐52. e45 32827525 10.1016/j.jpeds.2020.08.037 PMC7438214

[28] Butsch WS , Hajduk A , Cardel MI , et al. COVID‐19 vaccines are effective in people with obesity: a position statement from the Obesity Society. Obesity (Silver Spring). 2021. 10.1002/oby.23251 PMC8441899 34212511

